# Diaphragm Involvement in Heart Failure: Mere Consequence of Hypoperfusion or Mediated by HF-Related Pro-inflammatory Cytokine Storms?

**DOI:** 10.3389/fphys.2019.01335

**Published:** 2019-10-24

**Authors:** Jens Spiesshoefer, Matthias Boentert, Izabela Tuleta, Alberto Giannoni, Daniel Langer, Hans Joachim Kabitz

**Affiliations:** ^1^Institute of Life Sciences, Scuola Superiore Sant'Anna, Pisa, Italy; ^2^Respiratory Physiology Laboratory, Department of Neurology With Institute for Translational Neurology, University of Münster, Münster, Germany; ^3^Department of Cardiology I, University Hospital Muenster, Münster, Germany; ^4^Cardiology and Cardiovascular Medicine Division, Fondazione Toscana Gabriele Monasterio, National Research Council, CNR-Regione Toscana, Pisa, Italy; ^5^Respiratory Rehabilitation Unit, Respiratory Division, University Hospitals Leuven and Department of Rehabilitation Sciences, Leuven, Belgium; ^6^Department of Pneumology, Cardiology and Intensive Care Medicine, Klinikum Konstanz, Konstanz, Germany

**Keywords:** diaphragm disease, heart failure, systemic inflammation, basic science, lung function

**Graphical Abstract:**
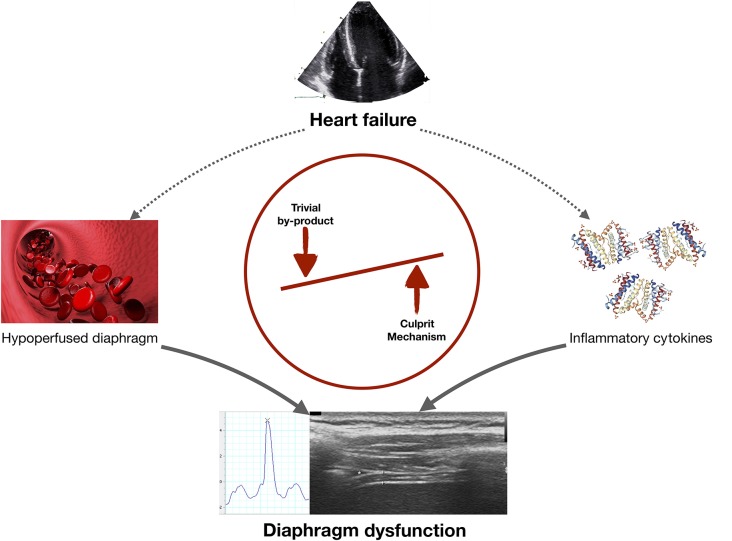
In heart failure (HF) diaphragmatic dysfunction may develop via two mechanisms: (i) diaphragm hypoperfusion could decrease diaphragmatic contractility (meaning that diaphragm dysfunction is a by-product of HF); or (ii) cirulating pro-inflammatory cytokines could directly alter diaphragm function (meaning that this potential mechanism should be investigated in future research projects).

## Introduction

Chronic heart failure (HF) has reached an epidemic extent and—through its high morbidity and mortality rates—imposes a remarkable burden on healthcare systems worldwide (Bleumink et al., 2004; Cowie, 2017). To date (mainly), two main HF entities have been defined, each at the two extremes of the spectrum of underlying pathophysiological disease mechanisms: (i) “systolic” HF (HF with reduced ejection fraction; HFrEF) and (ii) “diastolic” HF (HF with preserved ejection fraction; HFpEF) (with HF with midrange ejection fraction representing an intermediate condition between the two that has just been defined ~2 years ago) (Ponikowski et al., 2016). Much knowledge has been generated over recent decades about the pathophysiology of HFrEF, in particular. This has led to the pathophysiological notion that, in HFrEF, increased sympathetic drive, neurohormonal derangement and increased activity of the renin-angiotensin-aldosterone (RAAS) axis all develop in response to decreased cardiac output (i.e., systolic dysfunction) which is associated with pulmonary congestion and renal hypoperfusion (Floras, 2009; Ponikowski et al., 2016). This pathophysiological concept has been supported by major milestones in the pharmacological treatment of HFrEF, with all guidelines recommending use of agents that have proven mortality benefits in HFrEF based on activity linked to one or several of the above mentioned mechanisms (Ponikowski et al., 2016). In fact, according to the latest European Society of Cardiology guidelines (2016), HFrEF patients should now be prescribed pharmacological “triple therapy,” consisting of a beta blocker (e.g., bisprolol, target dose 10 mg o. d.), an angiotensin converting enzyme inhibitor (e.g., ramipril, target dose 10 mg o. d.) and a mineralocorticoid receptor antagonist (e.g., spirinolactone, target dose 50 mg o. d.) (Ponikowski et al., 2016; Cowie, 2017). Whereas low doses are recommended for treatment initiation, maintenance therapy aims for stable high doses in order to achieve the highest mortality benefit possible (Ponikowski et al., 2016). However, despite the advances in therapy for HFrEF that have been achieved over the last few decades, it appears that the number of breakthroughs in pharmacological therapy of HFrEF over the past 15 years is small. The only exception is a novel angiotensinreceptor-neprilysin inhibitor that is now part of guideline recommendations but still interferes with one of the well-known mechanisms of action (Neubauer, 2007; Ponikowski et al., 2016).

Dyspnea and exercise intolerance are among the clinical hallmarks of HF, but may be hard to be explained by cardiac dysfunction, hemodynamic and reflex system abnormalities alone (Meyer et al., 2001) and dysfunction of both skeletal and respiratory muscles may contribute to reduced exercise capacity in patients with HFrEF (Meyer et al., 2001).

## Diaphragmatic Dysfunction in HFrEF

The diaphragm is the key inspiratory muscle, with ~70% of the forced vital capacity during normal quiet breathing being achieved through proper diaphragm contraction. The force generated by this contraction causes pleural pressure to become more negative and abdominal pressure to be more positive (the difference is the transdiaphragmatic pressure, which increases during inspiration) (Mead et al., 1984). Initial descriptions of the involvement of the inspiratory muscles in HFrEF date back to the 1990s (Mancini et al., 1992). Mancini and colleagues showed that patients with HFrEF (*n* = 10) had lower maximum inspiratory pressure (PImax) compared with healthy controls (Mancini et al., 1992). Notably, a strong and significant correlation was shown between ratings of perceived dyspnea and PImax values underlying the potential clinical importance of diaphragm dysfunction in HFrEF.

Using invasive measurement of the transdiaphragmatic pressure (“twitch” Pdi) following electrical or magnetic stimulation of the phrenic nerve roots it was later shown that patients with HFrEF exhibit impaired contractility of the diaphragm as compared to healthy individuals (Hughes et al., 1999). This diagnostic method is considered the gold standard of respiratory muscle strength testing since it overcomes most of the technical flaws associated with volitional tests such as FVC or PImax (Laveneziana et al., 2019; Spiesshoefer et al., 2019). Furthermore, diaphragm ultrasound has emerged as a novel tool for assessment of diaphragm function. Specifically, the diaphragm thickening ratio (DTR) may reflect diaphragm strength (Cardenas et al., 2018) and has (by application of this methodology) been shown to be impaired in HFrEF patiens too (Caruana et al., 2001; Miyagi et al., 2018). Subsequently, Meyer and colleagues reported in a larger cohort of HFrEF patients (*n* = 244) that, approximately every third patient showed abnormal PImax values, which were associated with a worse overall prognosis (Meyer et al., 2001). Notably, this study showed that PImax adds prognostic value beyond known risk factors of clinical deterioration, including peak oxygen consumption, left ventricular ejection fraction, and norepinephrine plasma concentration (Meyer et al., 2001). Again, the clinical significance of diaphragm (inspiratory muscle) dysfunction has been underlined by strong and significant correlations obtained between PImax values and NYHA functional class and PImax and peak oxygen consumption. These findings have been extended over the last 15 years (Caruana et al., 2001; Meyer et al., 2001; Bowen et al., 2015). However, PImax is a volitional test, and results are hence highly depend on patient effort, leading to substantial intra-individual variability. Specifically, if PImax values are systematically lower in a particular patient cohort than in control subjects this may just reflect that patients are possibly less cooperative than healthy volunteers. Therefore, correct supervision and multiple repetition of the tests are both essential, as highlighted in the current European Respiratory Society statement on respiratory muscle strength testing (Laveneziana et al., 2019).

But does diaphragm dysfunction really matter in HFrEF or is it just a by-product of systolic ventricular dysfunction?

In a highly elaborate study, Mancini and collegues, were able to indirectly show that respiratory muscle ischemia is present in HFrEF patients, resulting in a higher prevalence and greater extent of low-frequency diaphragmatic fatigue which is failure to maintain a given diaphragmatic pressure at low phrenic nerve stimulation rates (i.e., at 1–20 Herz; Mancini et al., 1992) (in contrast to this high-frequency diaphragmatic failure that is characterized by inability to maintain a given diaphragmatic pressure at high rates of phrenic nerve stimulation: i.e., at 50–100 Herz). This finding provides a first, intuitive mechanistic explanation of diaphragm dysfunction in HFrEF. Of note, this would make treatment of (underlying) systolic HF *per se* through known pharmacological and interventional therapeutic pathways the primary treatment of (consecutive) diaphragmatic dysfunction in HFrEF.

Therefore, the key question is, are there mechanisms underlying diaphragmatic dysfunction in HFrEF that go beyond hypo-perfusion?

In a minipig animal model of HFrEF, Howell and colleagues not only confirmed the hypothesis that HFrEF leads to reduced diaphragmatic contractility but also showed a shift in diaphragmatic fibers from type IIa (fast twitch type) to type I (slow twitch type) (Howell et al., 1995). While these histological alterations seen in the diaphragm of HFrEF in animal models are intriguing at first, this may also just be a maldaptive response to underperfusion too.

Circulating pro-inflammatory cytokines have been hypothesized to comprise another mechanistic link between HFrEF and diaphragmatic dysfunction (see Graphical Abstract). It is now well established that patients with severe HFrEF show increased levels of circulating pro-inflammatory cytokines (interleukin [IL]-1-beta, IL-6 and tumor necrosis factor [TNF]-α in particular), and these my even impact overall functional status and prognosis (Torre-Amione et al., 1996; MacGowan et al., 1997; Rauchhaus et al., 2000; Janssen et al., 2005; van Tassell et al., 2012). Of note, these cytokines (TNF-α and IL-6 in particular) have been shown to directly impair muscle function in animal models (Gosselin et al., 2003; Janssen et al., 2005). In particular, Gosselin and coworkers proved the concept that there is a causal, direct association between circulating TNF- α and diaphragm dysfunction (Gosselin et al., 2003). In an animal model of dystrophic mice the impact of deletion of TNF- α on ventilatory function, diaphragm contractility and myosin heavy chain distribution was investigated (Gosselin et al., 2003). Thereby it was shown that elimination of TNF- α significantly improved diaphragm muscle maximal isometric force and that (in the long run) altered myosin heavy chain isoform profile of the diaphragm was detectable (Gosselin et al., 2003).

Some previous experimental works have helped clear the fog around the pathophysiology of diaphragm dysfunction in HF. First. using a a rat model of HFrEF of experimentally-induced HFrEF (ligation of the left coronary artery) it was recently shown that heart failure is associated with increases in circulating levels of TNF-α, IL-1-beta, and IL-12 (Seiler et al., 2016). Diaphragmatic function was postulated to remain largely unaffected—however, this was not assessed using gold-standard methods. Second, using a rat model of HFrEF too, the intracellular signaling pathways involved in skeletal mysoin heavy chain isoform alteration in the diaphragm induced by HF have been highlighted (Lima et al., 2014). Therein it was not only shown that HF induces a shift in diaphragmatic fibers from type IIa (fast twitch type) to type I (slow twitch type) but also that TNF- α serum concentration positively correlates with this shift in diaphragmatic fibers (Lima et al., 2014). Notably, it has been further highlighted that different intracellular pathways regulate diaphragm muscle expression of myosin heavy chain isoforms (and hence its change in expression): as such especially the mitogen-activated protein kinase family and therein the extracellular signal regulated kinase 1/2 play a decisive role in myosin heavy chain isoform maintenance (Chen et al., 2007; Lima et al., 2014). Combined with the *in vitro* finding that mitogen-activated protein kinase can be modulated by inflammatory cytokines (particularly TNF- α) and oxidative stress it can therefore be stated that there is experimental evidence speaking out in favor of inflammatory cytokines (particularly TNF- α) linking HF and diaphragm alterations and potentially dysfunction (Chen et al., 2007). While for TNF-α such a model is to the best of our knowlegde still missing an elegant experiment has proven the causal role of oxidative stress in mediating diaphragm dysfunction in HF (Ahn et al., 2015): therein it was shown that in a knock out model of p47phox-dependent Nox2 isoform of NAD(P)H oxidase (a putative source of diaphragm oxidants) experimentally induced HFrEF diaphragm isometric force, shortening velocity, and peak power were decreased by 20–50% in HF wild-type mice whereas p47phox knockout mice were protected from impairments in diaphragm contractile function elicited by HF. This finding is in accordance with animal expirmental longitudinal data that succesfully showed that administratin of the antioxidant N-acetyl-cysteine attenuates functional and structural (diaphragm) muscle alterations (Barreiro et al., 2016).

Further mechanisms likely underlying diaphragm dysfunction in HF that go beyond ciruclating pro-inflammtory cytokines and oxidative stress have recently been elegantly and more comprehensively than in the present dedicated opinion article reviewed by Kelley and Ferreira (2017). In short the point was made that increased sympathetic nerve activity, sphingolipid signaling, increased proteolytic activity and potentially HF mediated changes in the neuromuscular junctions, the excitation-contraction coupling and the contractile apparatus play further roles in the undoubtedly complex and multifactorial pathophysiology of diaphragm dysfunction in HF (Kelley and Ferreira, 2017).

However, to date, this issue has not been comprehensively studied in humans at all.

Inspiratory muscle training in HFrEF patients has been shown to have favorable effects on dyspnea and functional capactiy (Bosnak-Guclu et al., 2011; Marco et al., 2013; Beeler et al., 2014). Plus, it has recently been elegantly reviewed that inspiratory dysfunction in HF is unaffected by acute decompensation of heart failure and is not reversed by heart transplant (Kelley and Ferreira, 2017). These findings are considered to be two further indicators of the importance of impaired diaphragmatic function in HFrEF implying that in HF mechanisms beyond cardiovascular abnormalities *per se* are responsible for diaphragm dysfunction.

## Diaphragmatic Dysfunction in HFpEF

Less is known about diaphragmatic dysfunction, its pathophysiological role and clinical implications in patients with HFpEF. Over ~ the last 10 years, HFpEF has been among the most studied topics in cardiovascular research. It is now accepted that HFpEF is highly prevalent worldwide and is associated with morbidity and mortality rates comparable to those of HFrEF, meaning that HFpEF also places a major burden on healthcare systems worldwide (Bleumink et al., 2004; Cowie, 2017). However, unlike in HFrEF, no guideline-recommended treatment that would favorably alter clinical symptoms and prognosis is available for HFpEF, to date (Ponikowski et al., 2016).

The understanding of diaphragmatic involvement and its underlying mechanisms is still in its infancy in HFpEF. Recent animal and clinical studies have proven the concept that molecular, mitochondrial, histological and functional alterations in the diaphragm are associated with HFpEF, and that these are partly reversible by exercise training (Bowen et al., 2015; Seiler et al., 2016; Yamada et al., 2016). As in HFrEF a close association of dipahragm function (diaphragm thickening ratio as and intutive –ultrasound derived- metric reflecting the diaphragms capacity to contract) with exercise intolerance (as expressed by the 6 min walking distance achieved) was shown in HFpEF patients confirming its clinical importance therein too (Yamada et al., 2016). Again, in an animal model (high salt diet), Seiler and collegues showed that HFpEF is associated with increased circulating levels of IL-1-beta and IL-12, in particular (Seiler et al., 2016). While the point was made (as for HFrEF) that diaphragm function remained largely unaffected, the same limitations mentioned above still hold (diaphragm function was not quantitatively evaluated). The decisive role of plasma markers of inflammation has also been postulated and aknowledged from a translational perspective by Paulus and Tschöpe lately (Paulus and Tschöpe, 2013).

## Future Research

In order to elucidate to what extent inflammatory processes contribute to diaphragm dysfunction in heart failure of different etiology, future clinical research is needed. It is desirable to combine non-volitional techniques of respiratory muscle strength testing (i.e., twich transdiaphragmatic pressure and diaphragamatic ultrasound; Laveneziana et al., 2019) with quantification of cardiac function and exercise intolerance and detailed laboratory analyses including circulating pro-inflammatory cytokines and natriuretic peptides. Such a multimodal approach would help dissect the complex pathophysiology of dyspnoea and exercise intolerance and help clarify the mechanisms that underly diapragmatic dysfunction in HF. These mechanisms may even be interrelated among themselves (cardiac dysfunction, systemic and diaphragm hypoperfusion, increase in circulating proinflammatory cytokines and resulting diaphragm dysfunction). Therefore, an animal model tying onto the findings of Seiler and colleagues would be a decisive potential next step. Ideally this animal model would assess diaphragmatic function by gold-standard techniques as mentioned above and assess it over time after TNFα depletion to prove a causal link between TNFα and diaphragm function in HF (as has been succesfully done in dystrophic mice). Ultimately it would be desirable to conduct such animal experimantal work both in animal models of HFrEF and HFpEF (separetely) as the cytokine profile seen in HFpEF may differ from HFrEF and the effects on diaphragm function may hence be different in these two HF entitities (Seiler et al., 2016).

## Conclusion

In HFrEF, diaphragmatic dysfunction likely contributes to exercise intolerance, dyspnea and impaired overall prognosis, beyond what can be explained from indices of heart function and neurohormonal derangement alone. However, much remains unknown about the pathophysiology underlying diaphragm dysfunction in HF, especially in HFpEF. Diaphragm dysfunction in HF is either just a “by-product” (if it occured only due to hypoperfusion) or could also be related to systemic inflammation. Therefore, future research needs to focus on accurate and comprehensive measurement of diaphragm function and its association with pro-inflammatory cytokines in patient with HFrEF and HFpEF. This may be a mechanism that could be targeted to preserve diaphragmatic function and potentially improve exercise intolerance in HFrEF and HFpEF.

## Author Contributions

All authors listed have made a substantial, direct and intellectual contribution to the work, and approved it for publication.

### Conflict of Interest

The authors declare that the present manuscript was written in the absence of any commercial or financial relationship that could be considered as a potential conflict of interest. However, outside the present work JS is supported by the Else-Kröner-Fresenius Stiftung (Grant A109), by the Kommission für Innovative Medizinische Forschung an der Medizinischen Fakultät Muenster (IMF Grant SP 11 18 15) and by Deutsche Herzstiftung (DHS S/01/19) for basic respiratory physiology work related to heart failure. MB is supported by Sanofi-Genzyme outside this work and has received speaker honoraria and travel grants from Sanofi-Genzyme and Löwenstein Medical. IT and AG report not to have conflicts of interest in the context of the present manuscript. HK was supported by Deutsche Forschungsgemeinschaft (DFG) outside this work and received travel grants and/or speaking fees outside the submitted work from Löwenstein Medical, Weinmann, Philips Respironics, ResMed, Vivisol, Sapio Life and Sanofi-Genzyme.
